# Managing cataract surgery in patients with uveitis

**Published:** 2019-02-10

**Authors:** Aravind Harapriya, Eliza Anthony

**Affiliations:** 1 Head of Cataract Services: Cataract and IOL Services, Aravind Eye Hospital, Chennai, India.; 2 Consultant: Medical Consultant Uvea Services, Aravind Eye Hospital, Chennai, India.

**Cataract surgery in patients with uveitis has multiple challenges, of which control of inflammation is the most important.**

Long-standing intraocular inflammation (uveitis) leads to the formation of cataract (Figure 1), as does prolonged steroid use. There are multiple surgical challenges in managing uveitic cataract, such as the presence of posterior synechiae, atrophic iris, small pupil, pupillary membrane, fibrous anterior capsule, mature cataract, new vessels in the angle and zonular weakness. Postoperative inflammation, macular oedema and glaucoma are also more frequent in uveitic cataracts. This article will address these issues, but probably the most important issue of all is the control of inflammation preoperatively and its role in determining the surgical and visual outcome.

**Figure 1:**
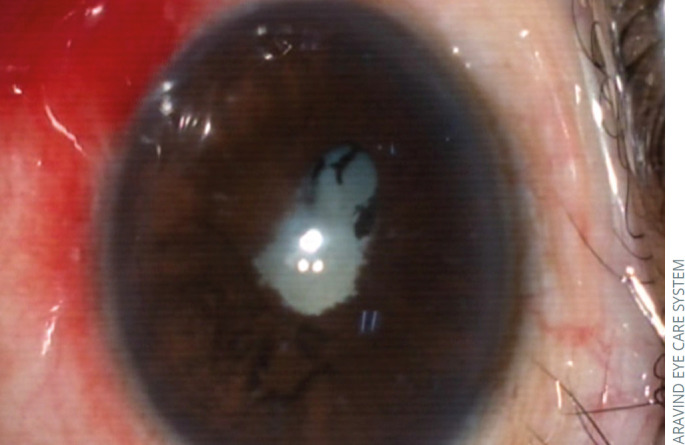
Cataract in a patient with uveitis

## Preoperative control of inflammation

As uveitis is commonly associated with systemic conditions, it is important to control systemic inflammation, if present. This frequently requires a multidisciplinary approach. It is necessary to have at least 3 months of quiescence (stable treatment without signs or symptoms of inflammation) in order to have a successful surgical outcome.

Uveal inflammation is said to be controlled when there is no vitritis and no cells in the anterior chamber. A small amount of residual flare is quite common, even if the inflammation is well controlled, as long-standing inflammation leads to the breakdown of the blood-aqueous barrier.

Surgery for uveitic cataract must take place under steroid cover. Start the patient on 1 mg/kg/day of oral prednisolone at least one week before surgery. An alternative strategy would be to intensify the use of topical steroid for 1–2 weeks before the operation. For example, give prednisolone eye drops 1% eight times a day for ten days and then, on the day of surgery, give an intravenous infusion of 500 mg methylprednisolone in 100 ml normal saline over 45–60 minutes. In more significant inflammation, consider immunosuppressive drugs. Some surgeons also prefer to start topical non-steroidal anti-inflammatory drugs (NSAIDs) one week before surgery.

A thorough retinal and posterior segment examination is necessary to look for macular oedema, optic atrophy, macular scarring, choroidal neovascular membrane and epiretinal membrane. Retinal complications can cause poor visual outcomes and often need to be treated preoperatively. In patients with mature cataract and dense posterior subcapsular cataract, ultrasonography is a useful tool to detect vitritis, the presence of retinochoroidal complex thickening, exudative retinal detachments and disc oedema.

In well-resourced settings, optical coherence tomography of the retina can help to document and monitor macular pathologies before surgery. Ultrasound biomicroscopy helps to assess pars planitis, uveal effusions, ciliary body traction, ciliary body atrophy and the presence of cyclitic membranes, especially in patients with hypotony. Some surgeons use a potential acuity meter to measure the potential improvement in visual acuity in patients with advanced cataract.

### Uveitic glaucoma

Raised intraocular pressure and glaucoma can be caused by uveitis or prolonged steroid use. Resist the temptation to combine cataract surgery with a drainage procedure. The failure rate for the drainage surgery will be very high, and the chance of subsequent successful glaucoma surgery will be reduced.

## Intraoperative management

The type of anaesthesia depends on the surgeon's preference, local factors and the presence of posterior synechiae. Surgery can occasionally be performed under topical anaesthesia; for example, in patients with posterior subcapsular cataract associated with Fuch's uveitis. However, if iris manipulation is required, then retrobulbar, sub-Tenon's or peribulbar blocks are preferable.

Both manual small-incision cataract surgery and phacoemulsification are found to be comparable in terms of endothelial cell loss and complication rates.^1^ Careful consideration of surgical strategy is essential if there is any sign of corneal endothelial dystrophy, or if phacoemulsfication is being used with a dense cataract (pp. 86–87).

**Figure 2:**
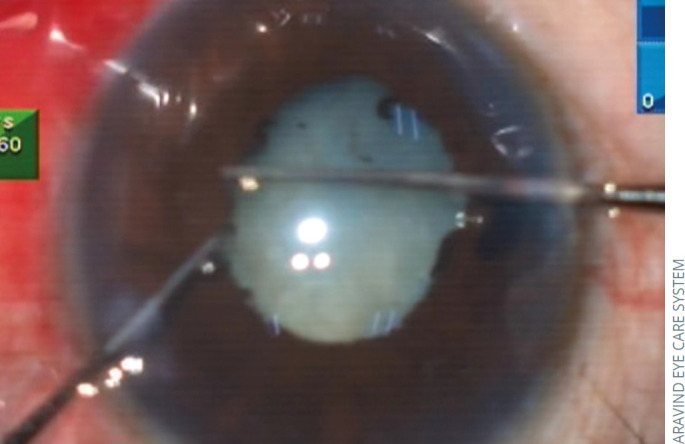
Intraoperative mechanical dilation of the pupil using Kuglen's hooks

Small pupil is a big challenge when operating on uveitic cataract. Pupillary membranes can be transected with capsulorrhexis scissors, and then removed with capsulorrhexis forceps prior to mechanical dilation of the pupil using Kuglen's hooks (Figure 2) or similar devices (pp. 84–85).

**Figure 3:**
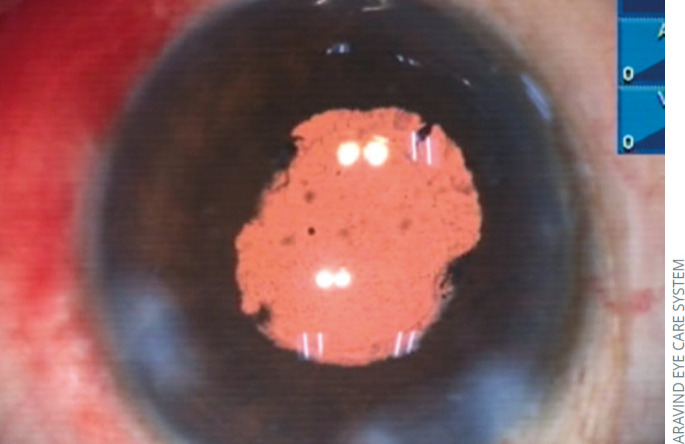
End of the procedure with PCIOL placed in bag

Iris manipulation must be done gently, as excessive manipulation can increase pigment dispersion, postoperative inflammation and hyphaema, and can result in permanent pupillary dilation. We recommend doing a large capsulorrhexis, because postoperative anterior capsular phimosis is more frequent in uveitic eyes. It is preferable to place the posterior chamber intraocular lens (PCIOL) in the bag (Figure 3), and not in the sulcus, in order to prevent postoperative iris irritation.^2^

### Postoperative capsule contraction and IOL selection

Capsule contraction after surgery can lead to anterior capsular phimosis, zonule rupture and, in severe cases, even lens dislocation. Polymethylmethacrylate (PMMA) intraocular lenses (IOLs), or foldable IOLs with PMMA haptics, minimise capsule contraction compared to IOLs with prolene haptics. Capsular tension rings also help to prevent contraction.

The choice of IOL is important for other reasons also:

- Hydrophobic acrylic IOLs are well tolerated in uveitic eyes and reduce the rate of posterior synechiae formation.^3^
- Heparin surface modified IOLs (HSM-IOLs) may be associated with a reduced rate of postoperative uveitis. Angulated IOL haptics help to reduce the amount of contact between the iris and the optic.^4^
- In aggressive uveitis, such as Behcet's and juvenile rheumatoid uveitis, inserting an IOL during cataract extraction has shown only moderate success and aphakia should be considered, especially in younger age groups.

In Fuchs' uveitis and non-granulomatous inflammation, inserting an IOL during the primary procedure is shown to be safer.^5^

Postoperative vitreous opacification is another frequent complication in uveitic eyes. Remove the cataract first and later, after assessing the impact of vitreous opacification on vision, decide whether or not to perform pars-plana vitrectomy.^5^

**Figure:**
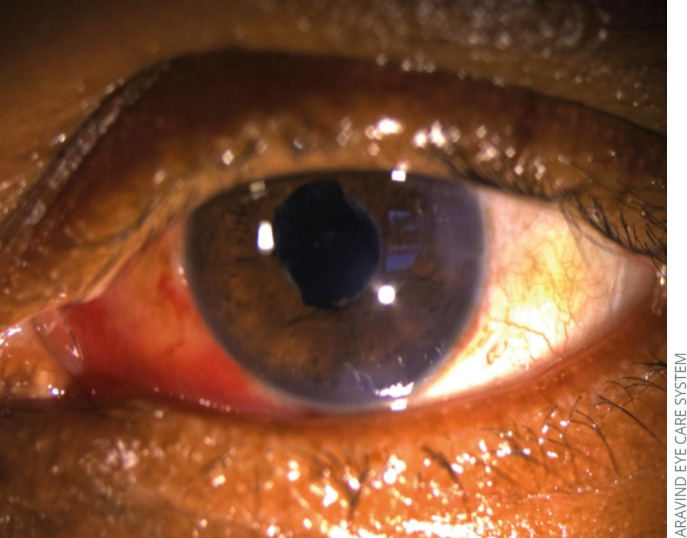
Image of the patient two days after surgery.

## Postoperative management

Aggressive control of inflammation is recommended after cataract surgery in uveitic eyes. Instil topical steroids hourly at first, and then taper the dose as per the response. Cycloplegics can be added for the first two weeks, as they reduce the ciliary spasm, stabilise the blood-aqueous barrier and prevent the formation of posterior synechiae.

Intraocular pressure rise, due to steroid response and inflammation, must be monitored and treated with topical antiglaucoma medications. Prostaglandin analogues should be avoided as they increase inflammation. Beta blockers, and topical and oral carbonic anhydrase inhibitors, can be safely used to manage the IOP spikes. Postoperative iris bombe (apposition of the iris to the IOL or anterior vitreous, preventing aqueous from flowing from the posterior to the anterior chamber), due to occlusio or seclusio pupillae, is treated with YAG laser peripheral iridectomy.

Other complications which lead to reduced vision are cystoid macular oedema, epiretinal membranes (ERM) and posterior capsular opacification.^6^ Cystoid macular oedema can be treated with topical steroids and NSAIDs at first. In unresponsive cases, we recommend posterior sub-Tenon's injection of triamcinolone (up to 40 mg in 1 ml) or intravitreal steroid preparations. Late complications, such as a thick epiretinal membrane, can cause significant pucker and visual deterioration. These patients can be referred to a vitreoretinal surgeon for an ERM peel. Postoperative cyclitic membranes and hypotony require pars plana vitrectomy and membranectomy. Another late complication is posterior capsular opacification which can be treated with YAG capsulotomy several months after the operation, once the inflammation is quiescent.

In paediatric uveitic cataract, visual outcome can be poor due to amblyopia and recalcitrant inflammation.

In conclusion, managing cataract in patients with uveitis is a challenge. However, with good preoperative assessment and adequate control of inflammation, good surgical visual outcomes can be achieved. Speak to the patient at every step about the visual prognosis, the pros and cons of treatment, and the risks factors associated with surgery. This adds to patients' satisfaction – which is of prime importance to the operating surgeon.
